# *Mycobacterium heraklionense* sp. nov.: A case series

**DOI:** 10.3892/etm.2015.2683

**Published:** 2015-08-14

**Authors:** IOANNIS K. NEONAKIS, DEMETRIOS A. SPANDIDOS, ZOE GITTI

**Affiliations:** 1 Department of Clinical Bacteriology, Parasitology, Zoonoses and Geographical Medicine, University Hospital of Heraklion, Heraklion, Crete, Greece; 2 Department of Laboratory Medicine, School of Medicine, University of Crete, Heraklion, Crete, Greece

**Keywords:** *Mycobacterium heraklionense*, tenosynovitis

## Abstract

*Mycobacterium heraklionense* sp. nov. (*M. heraklionense*) is a novel non-tuberculous mycobacterium belonging to the *Mycobacterium terrae* complex that has recently been described. It has a world-wide distribution. Recently, a case of tenosynovitis in an immunocompetent individual caused by *M. heraklionense* was reported, indicating that it has the ability to cause diseases. In the present study, in order to provide a more detailed profile of this mycobacterium and to obtain a more complete overall picture of its clinical significance, we report all available data regarding the initial 12 cases of its isolation. Of the 12 patients, 5 (42%) eventually died within a period of 3 months following the isolation of the mycobacterium. However, any connection between the presence of *M. heraklionense* and these deaths could not be documented. These 5 patients were all males with a mean age of 74.6 years suffering from serious underlying diseases, which most probably were the cause of death. Additional data from possible new cases of *M. heraklionense* isolation are anticipated.

## Introduction

The incidence of diseases caused by non-tuberculous mycobacteria (NTM) has dramatically increased over the past decades, due to the AIDS epidemic and the increase in the number of immunocompromised patients (1–4). The *Mycobacterium terrae* complex (MTC) is a group of NTM that includes non-pigmented, slow-growing organisms, usually found in soil. Members of this complex are *M. triviale*, *M. nonchromogenicum*, *M. hiberniae*, *M. arupense*, *M. senuense*, *M. kumamotonense* and *M. terrae* (5–8). They are uncommon colonizers of human epithelia and are generally regarded as non-pathogenenic (9). However, members of the MTC may cause human diseases, such as pulmonary infections, tenosynovitis, osteomyelitis, or even disseminated disease in HIV-infected patients (10–12).

*Mycobacterium heraklionense* sp. nov. (*M. heraklionense*) is a recently described non-tuberculous mycobacterium (13). Mature growth is obtained in solid media at temperatures between 25 and 37°C in 5–12 days. Colonies grow as smooth and unpigmented both in the dark and following light exposure. Isolates are susceptible to clarithromycin and resistant to quinolones, rifampicin, sulfamethoxazole and doxycycline. Phylogenetically, *M. heraklionense* is included in the MTC and is most closely related to *M. nonchromogenicum*. Some demographic and epidemiologic data regarding its first cases have been reported in its initial presentation (13).

In the present study, in order to obtain a more complete overall picture of its clinical significance, we report all available data regarding the initial 12 cases of *M. heraklionense* isolation.

## Case series

Twelve mycobacterial strains from different patients (designated as strains GN01 to GN12) were isolated from 2 outpatients and 10 inpatients, hospitalized at different medical wards of the University Hospital of Heraklion, the referral hospital in the island of Crete, Greece. The isolates could not be identified with conventional methods and were further analyzed with molecular techniques, including genetic sequencing, which revealed the presence of a new species designated as *M. heraklionense* (13).

A comparison of the phenotypic characteristics of *M. heraklionense* GN01 with those of the members of the MTC is shown in Table I. It should be noted that all isolates were analyzed with the GenoType commercial DNA strip assay, including GenoType Mycobacterium CM (Common Mycobacteria) and Additional Species (AS) commercial kits (Hain Lifescience GmbH, Nehren, Germany) in accordance with the manufacturer's instructions. All isolates yielded the same GenoType profile. Apart from the positive universal and mycobacterial genus strip bands, there were positive bands 10 and 12 when the GenoType CM and AS were used, respectively.

All available information regarding the samples along with the demographic and the epidemiological data of the patients is shown in Table II. The outpatient PB (GN05) had been hospitalized elsewhere a year ago due to active pulmonary tuberculosis (TB). In our hospital, he underwent bronchoscopy as a routine investigation of supraclavicular lymphadenopathy, with pulmonary lesions found in the CT scan. The data available for the other outpatient (GN07) were limited. The majority of the inpatients were elderly individuals with serious underlying diseases (Table II). Although none of them were HIV-positive, many of them had low lymphocyte counts. Five patients died within a period of 3 months following the isolation of the mycobacterium (patients HZ, PI, TS, KS and SK).

## Discussion

A drawback encountered in the present case series is that there was only one isolation of *M. heraklionense* per patient. This is due to the fact that the majority of clinicians in a number of hospitals worldwide tend to underestimate the potential hazards of NTM. They usually send only one sample per case and not three or more as they should do, when a possible mycobacterial infection is included in their differential diagnosis list. As a result, mycobacterial infections due to NTM cannot be firmly documented.

All strains were isolated within a 10-month period (December 2002 to Octomber 2003), with the great majority of them (75%; 9/12) isolated between July and September 2002 (Table II). This accumulation of cases cannot be explained, as no epidemiological link could be documented. In other words, there was no apparent correlation with independent parameters, such as the weather, a possible alteration in laboratory routine protocols, any construction works inside or in the vicinity of the hospital, etc.

As mentioned above, 2 isolates were derived from 2 outpatients. Furthermore 5 out of the remaining 10 patients were hospitalized for a period of ≤5 days prior to the isolation of *M. heraklionense*. These data strongly indicate that the infections of the patients did not occur during their hospitalization.

The vast majority of the patients were males (92%; 11/12). The mean age of the patients was 69.9 years and the mean hospitalization prior to the isolation of the mycobacterium was 17.5 days. Of the 12 patients, 10 were were of Hellenic nationality and only 2 (17%) were foreigners. This, along with the fact that the initial 4 cases were patients of Hellenic nationality, strongly supports the notion that this novel mycobacterium was not imported from abroad. Furthermore, accumulative data over the past few years have strongly revealed the worldwide distribution of *M. heraklionense*. The mycobacterium has been detected in various countries, including Greece, Italy, India, USA and the Czech Republic (13–16).

Of the 12 patients, 5 (42%) eventually died within a period of 3 months following the isolation of the mycobacterium. These 5 patients were all males with a mean age of 74.6 years and a mean stay in the hospital prior to isolation of 8.2 days. All of them had serious underlying diseases, such as cardiopulmonary failure, congestive heart failure, chronic obstructive pulmonary disease, myelodysplastic syndrome or cancer. A connection between the presence of *M. heraklionense* and the eventual death of the 5 patients cannot be ruled out. However, the fact that all these patients were elders with serious underlying diseases and the fact that they all died relatively shortly after their admission to the hospital, indicates that most probably, the cause of their deaths was their underlying diseases. At this point, it should be noted that recently, a case of tenosynovitis in an immunocompetent individual caused by *M. heraklionense* was reported (15). This is an indication that the mycobacterium has the ability to cause disease similar to other members of the MTC (15). Furthermore, in another case, *M. heraklionense* was isolated from a fine-needle aspiration sample obtained from a 53-year-old woman, who presented to an orthopedic surgery clinic in 2014 with a right-medial soft-tissue ankle mass (16). The patient had previously undergone a repair of a transverse laceration of her right Achilles tendon after a gardening accident 20 years prior. Based on the aforementioned data, *M. heraklionense* appears to have a pathogenic potency. However, in order to obtain a more substantial profile of the extent of the clinical significance of this mycobacterium, additional data from possible new cases are warranted and are being anticipated.

## Figures and Tables

**Table I.** Comparison of the phenotypic characteristics of *Mycobacterium heraklionense* GN01 with those of the members of *M. terrae* complex (5–8,17).

	Test results							
	
Species	Pigmentation	Niacin production	Nitrate reduction	Urease production	Aryl. 3 days	Glu	Catal. 68°	T80
GN01	–	–	+	–	–	+	+	+
*M. nonchromogenicum*	–	–	–	–	–	–	+	+
*M. terrae*	–	–	+	–	–	–	+	+
*M. triviale*	–	–	+	–	–	–	+	+
*M. kumanotonense*	–	–	+		–			+
*M. arupense*	–	–	–	–	–		+	+
*M. hiberniae*	–	–	+		–			
*M. senuense*	–	–	+	–	–		+	+

+, positive; −, negative; Aryl., arylsulfatase; Glu, β-glucosidase; Catal., catalase; T80, Tween-80 hydrolysis.

**Table II.** Demographic and epidemiologic data of the 12 patients.

	GN01^T^	GN02	GN03	GN04	GN05	GN06	GN07	GN08	GN09	GN10	GN11	GN12
Patient	LI	HZ	PI	PP	PB	ZI	MK	DG	ME	TS	KS	SK
Date of isolation (date/month/year)	16/12/02	19/2/03	14/7/03	22/7/03	2/8/03	8/8/03	8/8/03	13/8/03	21/8/03	8/9/03	10/9/03	31/10/03
Age (years)	74	76	62	83	42	70	–	59	35	82	76	77
Gender	M	M	M	M	M	M	F	M	M	M	M	M
Nationality	H	H	H	H	O	H	H	H	O	H	H	H
Sample	SP	SP	SP	SP	BAL	SP	U	SP	SP	SP	SP	SP
Ward	PN	HEM	PN	GAS	–	PN	–	HEM	PN	ICU	PN	ORTH
Compartment	C	C	C	A	–	C	–	C	C	C	C	B
Prior hospital stay	51	11	17	11	–	2	–	66	4	3	5	5
Underlying disease	D, RF, CHF	MDS	COPD	COPD, AN	TB	CA, COPD	–	NHL	REYM	CHF, PF	CPF	CA, COPD
Death within 3 mo.	N	Y	Y	N	N	N	N	N	N	Y	Y	Y

M, male; F, female; H, Hellenic; O, other; SP, sputum; BAL, broncho-alveolar lavage; U, urine; PN, pneumonology; Hem, hematology; GAS, gastroenterology; ICU, intensive care unit; ORTH, orthopedics; D, diabetes; RF, renal failure; CHF, congestive heart failure; MDS, myelodysplastic syndrome; COPD, chronic obstructive pulmonary disease; AN, anemia; TB, tuberculosis; CA, cancer; NHL, Non-Hodgkin lymphoma; REYM, rheumatologic disease; PF, pulmonary fibrosis; CPF, cardiopulmonary failure; Y, yes; N, no; mo., months.
